# Determinants of fertility differentials in Burundi: evidence from the 2016-17 Burundi demographic and health survey

**DOI:** 10.11604/pamj.2021.38.316.27649

**Published:** 2021-03-30

**Authors:** Jean Claude Nibaruta, Noureddine Elkhoudri, Mohamed Chahboune, Milouda Chebabe, Saad Elmadani, Abdellatif Baali, Hakima Amor

**Affiliations:** 1 Hassan First University of Settat, Higher Institute of Health Sciences, Health Science and Technology Laboratory, Settat, Morocco,; 2 Hassan First University of Settat, Higher Institute of Health Sciences, Department of Health Sciences, Settat, Morocco,; 3 Cadi Ayyad University of Marrakech, Semlalia Faculty of Science, Department of Biology, Marrakech, Morocco

**Keywords:** Fertility, demographic, factors, health survey, Burundi

## Abstract

**Introduction:**

although fertility control remains a major priority for the Burundian government and most of its partners, few studies on Burundi´s fertility determinants are available to guide interventions. To address this gap, our study aims to examine the most factors influencing fertility differentials in Burundi by using the latest Burundi demographic and health survey data.

**Methods:**

using data from the 2016-17 Burundi demographic and health survey, one-way analysis of variance was performed to describe variations in mean number of children ever born across categories of correlate variables. Then univariable and multivariable poisson regression analyses were carried out to identify the most factors influencing fertility differentials in Burundi.

**Results:**

in our sample, the total number of children ever born ranged from 0 to 15 children by women with a mean number of 2.7 children (±2.8 SD). Factors such as urban residence (aIRR 0.769, 95% CI: 0.739 - 0.782, p = 0.008), increase in the level of education of both women and husbands (aIRRs of 0.718, 95% CI: 0.643 - 0.802, P<0.001 and 0.729, 95% CI: 0.711 - 0.763, p<0.001 respectively), no history of infant mortality experience (aIRR 0.722, 95% IC: 0.710 - 0.734, p<0.001) and increase in age at first marriage or first birth (aIRRs of 0.864, 95% CI: 0.837 - 0.891, P<0.001 and 0.812, 95% CI: 0.781 - 0.845, p<0.001 respectively) are associated with a low fertility rate while factors such as residence especially in Southern region (aIRR 1.129, 95% IC: 1.077 - 1.184, p<0.001), women and husband´s agricultural profession (aIRRs of 1.521, 95% CI: 1.429 - 1.568, P<0.001 and 1.294, 95% CI: 1.211 - 1.316, p<0.001 respectively), household poverty (aIRR 1.117, 95% IC: 1.080 - 1.155, p<0.001), lack of knowledge of any contraceptive method (aIRR 1.502, 95% IC: 1.494 - 1.564, p<0.001) and non-use of modern contraceptive methods (aIRR 1.583, 95% IC: 1.562 - 1.607, p<0.001) are associated with a high fertility rate.

**Conclusion:**

the results of this study suggest that actions aimed at promoting education in general especially female education, improving child survival, women´s socio-economic status, agriculture mechanization and increasing number and scope of family planning services, could help reduce Burundi fertility rate.

## Introduction

**Figure 1:**
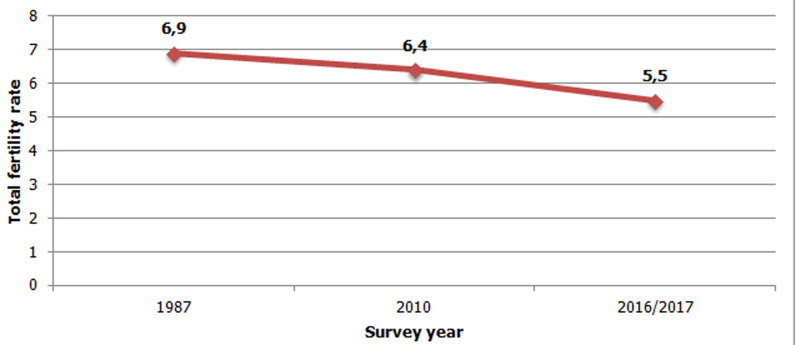
trends of fertility in Burundi, (data sources): 1987, 2010 and 2016-17 Burundi demographic and health survey reports

Several studies indicate that sub-Saharan Africa (SSA) countries are in demographic transition [1,2]. Some countries in this region are slow to engage in this transition and their fertility rate remain high [3,4]. Burundi is one of those countries where fertility rate is still high and one of the most densely populated countries in the world [5,6]. In Figure 1, we used total fertility rates (TFR) available in three Burundi demographic and health survey (BDHS) reports [7-9] to determine fertility trends in Burundi. The TFR decreased from 6.9 in 1987 to 6.4 in 2010 and from 6.4 to 5.5 in 2016/17, a decrease of only 1.4 children per woman over approximately thirty years. Numerous consequences linked to this high fertility are observed throughout the country. These include land conflicts which account for 80% of complaints at the judicial level [10]. Burundi also ranks among the poorest countries in SSA: 64.9% of Burundians live below the national poverty line of 1.27 US dollars and 38.7% live in extreme poverty [11]. Several studies indicate that high fertility negatively affects mother and child well-being [12-14]. In Burundi, the maternal mortality ratio (MMR) is estimated at 334 maternal deaths per 100,000 live births and the infant mortality rate (IMR) at 47 deaths per 1,000 live births [7]. These MMR and IMR remain among the highest in SSA [15].

It is then important for the Burundian government to reduce the fertility rate due to limited resources of the country. To try reducing this high fertility some measures to promote family planning (FP) services were carried out [10]. However, beside the influence of FP services, several studies indicate that women´s fertility are influenced by various socio-economic [2,16], demographic, cultural and biological factors [17,18]. Socio-political conflicts [19,20] and local environment factors [21] can also influence women´s fertility. Although fertility control remains a major priority for Burundi and most of its partners [10,22,23], few studies on fertility are available to guide interventions. From 1987 to 2017, only three BDHSs [7-9] had already been conducted. The results of these surveys are limited to a few determinants of fertility and are fully descriptive. Therefore, they do not provide a better understanding of the factors influencing fertility differentials in the Burundian settings. To address this gap, our study aims to examine the most factors influencing fertility differentials in Burundi by using the latest BDHS data.

## Methods

**Data sources:** we used data from the latest demographic and health survey conducted in Burundi in 2016-2017 (2016-17 BDHS) [7]. To access this data, we submitted a brief description of this study to the DHS program and after one day the requested access was granted [24]. The 2016-17 BDHS is a nationally representative cross-sectional survey that was able to collect comparable demographic and health data on a sample of 17,269 women aged 15-49 years. This sample of 17,269 women of reproductive age was drawn using a two-stage stratified sampling procedure. A detailed description of this sampling process is presented in the final report of the 2016-17 BDHS [7]. During data collection, women aged 15-49 years were asked about their birth histories and this provided information on the total number of children ever born to each woman, which is considered a dependent variable in our study.

### Variables

**Dependent variable:** our dependent variable is the total number of Children Ever Born (CEB) to a female respondent in the 2016-17 BDHS. The total number of CEB is a measure of the reported number of children born to a woman up to the moment at which the data was collected [25]. Two approaches such as current and cumulative approaches are used to measure fertility. The current fertility approach is based on current fertility behavior. The TFR and general fertility rate (GFR) can be used to measure the current fertility [26] as they rely on current fertility behavior. On the other hand, the cumulative fertility approach considers past fertility history of CEB to each cohort of women by age. In this study, we used the total number of CEB as our dependent variable because the total number of CEB for women belonging to the cohort of 15 to 49 years reflects both current and past fertility behavior [27]. In addition, because this study applies a Poisson regression model that deals with count outcomes such as the total number of CEB, we found CEB to be a more suitable outcome variable.

**Independent variables:** based on a prior literature review, eighteen variables divided into three groups (socio-demographic, environmental and cultural) were selected for analysis. For socio-demographic variables, eleven variables such as women´s age, place of residence, health regions, women´s marital status, both women and husband´s education and profession, household wealth index, women´s religion and sex of the household head were selected. For environmental variables, two variables: exposure to family planning messages and infant mortality experience were considered. Finally, five cultural variables: age at first marriage or at first birth, knowledge of any contraceptive methods, modern contraceptive use and family size preference were selected for analysis. Details on these selected independent variables and their suitable categories are presented in Table 1, Table 1 (suite). It should be noted that the variables “health regions” and “occupation” were recoded to reduce their excessive number of categories. The variable «Health regions» had eighteen categories corresponding to the current eighteen Burundi provinces. Based on the 2010 BDHS final report [9], this variable was recoded into five categories as follows: 1) the northern region includes the provinces of Kayanza, Ngozi, Kirundo and Muyinga; 2) the central-east region includes the provinces of Muramvya, Gitega, Karusi, Ruyigi and Cankuzo; 3) the western region includes the provinces of Cibitoke, Bubanza and Bujumbura Rural; 4) the southern region includes the provinces of Mwaro, Bururi, Rumonge, Rutana and Makamba and finally province of Bujumbura Mairie, which forms a separate region given its urban specificity. Similarly, with reference to Zah's study [2], the “occupation” variable had thirteen categories but it was recoded into three categories as follows: 1) modern occupations are defined as individuals who work in commerce, industry, services, armed forces, transportation, administration, and clergy; 2) agricultural occupations include the following types of occupations: farmers, salesmen, manual workers and related workers; 3) unemployed were defined as individuals of working age who reported that they had no job activity in the six months preceding the survey. The advantage of this categorization is that it minimizes the variability in respondent´s response due to job transition [2]. The latter is very common in low-income countries such as Burundi.

**Table 1:** variations in mean number of children ever born by the selected socio-demographic, environmental and cultural variables

Variables categories	MCEB	SD	F	P value
N=17269	2.7	2.8		
**Sociodemographic variables**				
**Age group**				
15-19	0.07	0.26	5,204.89	<0.001
20-24	0.91	1.01		
25-29	2.30	1.51		
30-34	3.93	1.88		
35-39	5.06	2.23		
40-44	6.02	2.55		
45-49	6.55	2.77		
**Place of residence**				
Rural	2.84	2.84	255.89	<0.001
Urban	1.84	2.32		
**Health regions**				
Bujumbura Mairie	1.64	2.14	58.95	<0.001
North	2.87	2.72		
Central-East	2.65	2.73		
West	2.96	2.90		
South	2.77	3.01		
**Marital status**				
Single	0.12	0.50	4,930.02	<0.001
Married/living together	4.83	2.54		
Divorced/separated	3.21	2.30		
Widowed	4.12	2.38		
**Women's education**				
Illiterate	4.25	2.81	1,708.55	<0.001
Primary	2.51	2.58		
Secondary	1.15	1.62		
Higher	0.75	1.49		
**Husband's education**				
Illiterate	4.79	2.64	2,372.38	<0.001
Primary	3.86	2.39		
Secondary	2.96	2.12		
Higher	2.62	1.71		
Don't know/NA^1^	0.89	1.94		
**Women's occupation**				
Modern	1.81	1.22	525.40	<0.001
Agricultural	3.10	2.83		
Unemployed	1.04	2.04		
Others/don't know	0.59	1.47		
**Husband's occupation**				
Modern	3.52	2.30	2,703.70	<0.001
Agricultural	4.46	2.57		
Unemployed	4.23	2.48		
Others/Don't know/NA^1^	0.95	2.01		

**Note: NA:** not applicable; **NA^1^**: women who did not provide information about their husbands because of their current marital status (single/divorced/separated/widowed, N=7,488); **Exposure to FP message^2^**: obtained by combining the following four variables: heard family planning on radio, TV, newspaper/magazine or by text messages on mobile phone in last few months; **NA^3^**: not yet married or single women (N=5,967); **NA^4^**: still no births (N=5,910); **MCEB**: mean number of children ever born; **SD:** standard deviation; **F:** Fisher-Snedecor test of ANOVA

**Table 1 (suite):** variations in mean number of children ever born by the selected socio-demographic, environmental and cultural variables

Variables categories	MCEB	SD	F	P value
**Wealth index**				
Poorest	3.12	2.70	72.27	<0.001
Poorer	2.87	2.79		
Middle	2.83	3.00		
Richer	2.75	2.84		
Richest	2.07	2.54		
**Women's religion**				
No religion	4.04	2.71	10.17	<0.001
Catholic	2.67	2.73		
Protestant	2.76	2.93		
Adventist	2.97	2.77		
Muslim	2.50	2.47		
Others	2.51	2.53		
**Sex of the household head**				
Male	2.89	2.86	183.27	<0.001
Female	2.24	2.57		
**Environmental variables**				
**Exposure to FP messages^2^**				
No	2.74	2.81	2.68	0.102
Yes	2.66	2.78		
**Infant mortality experience**				
Yes	5.91	2.43	8,639.43	<0.001
No	1.90	2.24		
**Cultural variables**				
**Age at first marriage**				
≤ 15 years	5.07	2.63	5,044.57	<0.001
16 - 19 years	4.22	2.62		
≥ 20 years	3.73	2.33		
NA^3^	0.12	0.50		
**Age at first birth**				
≤ 15 years	5.01	2.65	5,637.98	<0.001
16 - 19 years	4.36	2.59		
≥ 20 years	3.92	2.34		
NA^4^	0.00	0.00		
**Knowledge of any contraceptive methods**				
Has knowledge	0.20	0.91	401.39	<0.001
No knowledge	2.79	2.80		
**Modern contraceptive use**				
Yes	2.54	2.84	422.91	<0.001
No	3.76	2.29		
≥ 4 children	3.00	2.85		
No numeric response	2.39	3.05		

**Note: NA:** not applicable; **NA^1^**: women who did not provide information about their husbands because of their current marital status (single/divorced/separated/widowed, N=7,488); **exposure to FP message^2^**: obtained by combining the following four variables: heard family planning on radio, TV, newspaper/magazine or by text messages on mobile phone in last few months; **NA^3^**: not yet married or Single women (N=5,967); **NA^4^**: still no births (N=5,910); **MCEB**: mean number of children ever born; **SD:** standard deviation; **F:** Fisher-Snedecor test of ANOVA

**Statistical analysis:** the data analysis was conducted using STATA MP Software, version 13. As the 2016-17 BDHS sample was obtained using a two-stage cluster sampling process, the data were first weighted using the STATA svyset command before any statistical analysis to restore the representativeness of the survey and to tell STATA to consider the sampling design when calculating standards errors. We first conducted a descriptive analysis to describe the socio-demographic characteristics of the sample. Then one-way analysis of variance (ANOVA) was performed to describe variations in mean number of children ever born (MCEB) across categories of the selected independent variables. To identify factors influencing fertility differentials, both univariable and multivariable Poisson regression analyses were performed. The correlates with a p-value ≤ 0.2 in the univariable analysis were included in the multivariable Poisson regression model offset by the natural logarithm of the women´s age. For ease of the results interpretation, the coefficients were exponentiated to yield adjusted Incident Rate Ratio (aIRR). In multivariable analysis, variables with p<0.05 were declared to be significantly associated with fertility differentials.

**Ethical considerations:** the 2016-17 BDHS protocol, consent forms, and data collection instruments were reviewed and approved by both the National Ethics Committee for the Protection of Human Beings Participating in Biomedical and Behavioral Research in Burundi and the Institutional Review Board of ICF International. Moreover, data were collected after taking informed consent and all information was kept confidential. For this study, permission was given by the DHS program to access the 2016-17 BDHS dataset after review of the submitted brief description of this study [24]. The data were treated with utmost confidentially.

## Results

**Socio-demographic characteristics of the sample:** the socio-demographic characteristics of the sample are summarized in Table 2. The weighted sample size was 17,269 women aged 15-49, with an average age of 28.26 years (SD = 9.48). The findings indicate that slightly more than two out of five women (41.1%) were in the 15-24 years range. Most of these women (87.1%) lived in rural areas and more than half (55%) lived in the northern and central-eastern health regions. Analysis of marital status revealed that 56.6% were officially married or in a common-law relationship and 34.6% were still single. Regarding education, slightly more than three out of four women (75.4%) were either illiterate or had only a primary school education and only 1.2% had a higher level of education. Regarding occupations, the results show that 77.5% of women worked in agricultural occupations and only 4.9% worked in modern occupations. Similarly, regarding wealth index, the findings indicate that about three out of five women (59.1%) were classified either in poorest/poor or middle categories. Our findings also indicate that most of women (57.3%) were Catholic.

**Table 2:** socio-demographic characteristics of the sample

Variables categories	Frequency (n)	Percentage(%)
**Age (years)**		
15-19	3,859	22.3
20-24	3,244	18.8
25-29	3,002	17.4
30-34	2,443	14.1
35-39	1,967	11.4
40-44	1,545	8.9
45-49	1,209	7.0
**Place of residence**		
Rural	15,037	87.1
Urban	2,232	12.9
**Health regions**		
Bujumbura Mairie	1,304	7.6
North	5,136	29.7
Central -East	4,365	25.3
West	2,686	15.6
South	3,779	21.9
**Marital status**		
Single	5,967	34.6
Married/living with partner	9,782	56.6
Divorced/separated	887	5.1
Widowed	634	3.7
**Education**		
No education	6,259	36.2
Primary	6,775	39.2
Secondary	4,020	23.3
Higher	215	1.2
**Occupation**		
Modern	849	4.9
Agricultural	13,386	77.5
Unemployed	2,563	14.8
Other/don´t know	471	2.7
**Wealth index**		
Poorest	3,310	19.2
Poorer	3,432	19.9
Middle	3,456	20.0
Richer	3,370	19.5
Richest	3,701	21.4
**Religion**		
No religion	172	1.0
Catholic	9,899	57.3
Protestant	5,948	34.4
Muslim	545	3.2
Adventiste	455	2.6
Others	250	1.4

**Variations in the mean number of children ever born by the selected independent variables:** in our sample, the total number of children ever born (CEB) ranged from 0 to 15 children by women with a mean of 2.7 children (±2.8 SD). Table 1 and Table 1 (suite) presents variations in the mean number of children ever born (MCEB) per woman by the selected socio-demographic, environmental and cultural variables. According to the results of the One-way analysis of variance (ANOVA), there are very significant variations (p<0.001) in the MCEB per woman according to the variables age, place of residence, regions, marital status, both women and husband´s education and profession, wealth index, religion, sex of the household head, infant mortality experience, age at first marriage or at first birth, knowledge of any contraceptive methods, modern contraceptive use and family size preference.

**Determinants of fertility differentials:** to identify factors associated with fertility differentials, univariable and multivariable poison regression analyses were performed and results are summarized in Table 3 and Table 3 (suite). In the univariable and multivariable analyses, fertility differentials are described by variations in incident rate ratios (IRR) across categories of each correlate variable compared to the reference category. An IRR value that is greater than one means that a given category has a higher fertility rate than that of the reference category, while that less than one implies lower fertility rate compared to the reference category. In the univariable analysis factors such as place of residence, regions, marital status, both women and husband´s education and profession, wealth index, religion, sex of the household head, exposure to FP messages, infant mortality experience, age at first marriage and at first birth, knowledge of any contraceptive methods, modern contraceptive use and family size preference met the minimum criteria (p <0.2) for inclusion in the multivariable analysis.

**Table 3:** results of the univariable and multivariable analyses of correlates of fertility differentials in Burundi

	Univariable analysis		Multivariable analysis	
	uIRR (95% CI)	P Value	aIRR (95%CI)	P Value
**Place of residence**				
Rural (RC)	1.000		1.000	
Urban	0.678 (0.631 - 0.730)	<0.001	0.769 (0.739 - 0.782)	0.008
**Health regions**				
Bujumbura Mairie (RC)	1.000		1.000	
North	1.652 (1.472 - 1.854)	<0.001	1.018 (0.970 - 1.067)	0.469
Central-East	1.530 (1.362 - 1.719)	<0.001	1.062 (1.012 - 1.114)	0.014
West	1.717 (1.524 - 1.933)	<0.001	1.094 (1.042 - 1.148)	<0.001
South	1.607 (1.428 - 1.809)	<0.001	1.129 (1.077 - 1.184)	<0.001
**Marital status**				
Single (RC)	1.000		1.000	
Married/living together	21.689 (19.189 - 24.515)	<0.001	2.124 (1.60 - 2.815)	<0.001
Divorced/separated	17.008 (15.008 - 19.275)	<0.001	1.659 (1.537 - 1.791)	<0.001
Widowed	20.389 (17.940 - 23.173)	<0.001	1.852 (1.715 - 2.000)	<0.001
**Women's education**				
Illiterate (RC)	1.000		1.000	
Primary	0.739 (0.718 - 0.760)	<0.001	0.940 (0.924 - 0.956)	<0.001
Secondary	0.303 (0.235 - 0.392)	<0.001	0.767 (0.735 - 0.801)	<0.001
Higher	0.264 (0.243 - 0.286)	<0.001	0.718 (0.643 - 0.802)	<0.001
**Husband's education**				
Illiterate (RC)	1.000		1.000	
Primary	0.896 (0.876 - 0.916)	<0.001	0.948 (0.931 - 0.965)	<0.001
Secondary	0.699 (0.671 - 0.727)	<0.001	0.876 (0.842 - 0.911)	<0.001
Higher	0.550 (0.503 - 0.600)	<0.001	0.729 (0.711 - 0.763)	<0.001
Don't know/NA^1^	0.276 (0.260 - 0.293)	<0.001	0.968 (0.748 - 1.254)	0.807
**Women's occupation**				
Modern (RC)	1.000		1.000	
Agricultural	1.173 (1.107 - 1.243)	<0.001	1.521(1.429 - 1.568)	<0.001
Unemployed	0.514 (0.450 - 0.587)	<0.001	0.970 (0.929 - 1.013)	0.172
Others/don't know	0.319 (0.237 - 0.429)	<0.001	1.022 (0.951 - 1.098)	0.553
**Husband's occupation**				
Modern (RC)	1.000		1.000	
Agricultural	1.192 (1.155 - 1.231)	<0.001	1.294 (1.211 - 1.316)	<0.001
Unemployed	1.168 (1.104 - 1.237)	<0.001	0.992 (0.944 - 1.042)	0.742
Others/don't know/NA^1^	0.372 (0.351 - 0.396)	<0.001	1.026 (0.961 - 1.096)	0.435
**Wealth index**				
Richest (RC)	1.000		1.000	
Richer	1.329 (1.257 - 1.406)	<0.001	1.011 (0.987 - 1.035)	0.376
Middle	1.287(1.217 - 1.360)	<0.001	1.054 (1.032 - 1.077)	<0.001
Poorer	1.316 (1.244 - 1.393)	<0.001	1.094 (1.067 - 1.121)	<0.001
Poorest	1.404 (1.330 - 1.482)	<0.001	1.117 (1.080 - 1.155)	<0.001

**Note: uIRR**: unadjusted incident rate ratio; **aIRR**: adjusted incident rate ratio; **RC**: reference category; **95% CI:** 95% confidence interval; **NA^1^, Exposure to FP Messages^2^, NA^3^ and NA^4^** have the same meanings as in Table 2, results adjusted for women's age and marital status

**Table 3(suite):** results of the univariable and multivariable analyses of correlates of fertility differentials in Burundi

	Univariable analysis		Multivariable analysis	
	uIRR (95% CI)	P Value	aIRR (95%CI)	P Value
**Women's religion**				
No religion (RC)	1 .000		1.000	
Catholic	0.727 (0.663 - 0.797	<0.001	0.976 (0.911 - 1.046)	0.491
Protestant	0.773 (0.703 - 0.850)	<0.001	1.002 (0.935 - 1.075)	0.951
Adventist	0.715 (0.629 - 0.813)	0.002	0.953 (0.878 - 1.035)	0.197
Muslim	0.840 (0.752 - 0.939)	<0.001	0.949 (0.876 - 1.028)	0.251
Others	0.717 (0.612 - 0.841)	<0.001	0.925 (0.840 - 1.018)	0.112
**Sex of the household head**				
Male (RC)	1.000		1.000	
Female	0.772 (0.744 - 0.800)	<0.001	0.969 (0.946 - 0.992)	0.009
**Exposure to FP Messages^2^**				
No (RC)	1.000		1.000	
Yes	0.967 (0.939 - 0.996)	0.025	1.000 (0.983 - 1.018)	0.994
**Infant mortality experience**				
Yes (RC)	1.000		1.000	
No	0.457 (0.446 - 0.469)	<0.001	0.722 (0.710 - 0.734)	<0.001
Age at first marriage				
≤ 15 years (RC)	1.000		1.000	
16 - 19 years	0.853 (0.828 - 0.879)	<0.001	0.967 (0.939 - 0.995)	0.023
≥ 20 years	0.675 (0.655 - 0.696)	<0.001	0.864 (0.837 - 0.891)	<0.001
NA^3^	0.037 (0.033 - 0.042)	<0.001	1.000 (omitted)	
Age at first birth				
≤ 15 years (RC)	1.000		1.000	
16 - 19 years	0.872 (0.839 - 0.906)	<0.001	0.900 (0.867 - 0.934)	<0.001
≥ 20 years	0.700 (0.674 - 0.727)	<0.001	0.812 (0.781 - 0.845)	<0.001
NA^4^	0.000 (0.000 - 0.000)	<0.001	0.000 (0.000 - 0.000)	<0.001
Knowledge of any Contraceptive methods				
Has knowledge (RC)	1.000		1.000	
No knowledge	1.736 (1.710 - 1.742)	<0.001	1.502 (1.494 - 1.564)	<0.001
**Modern contraceptive use**				
Yes (RC)	1.000		1.000	
No	1.646 (1.640 - 1.684)	<0.001	1.583 (1.562 - 1.607)	<0.001
**Family size preference**				
≤ 3 children (RC)	1.000		1.000	
≥ 4 children	1.196 (1.161- 1.231)	<0.001	1.059 (1.042 - 1.076)	<0.001
No numeric response	0.980 (0.868 - 1.106)	0.738	1.090 (1.034 - 1.150)	0.002

**Note: uIRR**: unadjusted incident rate ratio; **aIRR**: adjusted incident rate ratio; **RC**: reference category; 95% CI: 95% confidence interval; **NA^1^, Exposure to FP Messages^2^, NA^3^ and NA^4^** have the same meanings as in Table 2, results adjusted for women's age and marital status

In multivariable analysis, factors such as urban residence (aIRR 0.769, 95% CI: 0.739 - 0.782, p = 0.008), increase in the level of education of both women and husbands (especially a higher education level) (aIRRs of 0.718, 95% CI: 0.643 - 0.802, P <0.001 and 0.729, 95% CI: 0.711 - 0.763, p<0.001 respectively), female headed households (aIRR 0.969, 95% CI: 0.946 - 0.992, p = 0.009), no history of infant mortality experience (aIRR 0.722, 95% IC: 0.710 - 0.734, p<0.001) and increase in age at first marriage or first birth (especially an age ≥ 20 years) (aIRRs of 0.864, 95% CI: 0.837 - 0.891, P <0.001 and 0.812, 95% CI: 0.781 - 0.845 , p<0.001 respectively) are associated with a low fertility rate while factors such as residence especially in Southern region (aIRR 1.129, 95% IC: 1.077 - 1.184, p<0.001), both women and husband´s agricultural profession (aIRRs of 1.521, 95% CI: 1.429 - 1.568, P <0.001 and 1.294, 95% CI: 1.211 - 1.316, p<0.001 respectively), household poverty (especially household in “poorest” category) (aIRR 1.117, 95% IC: 1.080 - 1.155, p<0.001), lack of knowledge of any contraceptive methods (aIRR 1.502, 95% IC: 1.494 - 1.564, p<0.001), non-use of modern contraceptive methods (aIRR 1.583, 95% IC: 1.562 - 1.607, p<0.001) and a number of children ≥ 4 as a family size preference (aIRR 1.059, 95% IC: 1.042 - 1.076, p<0.001) are associated with a high fertility rate.

## Discussion

Our study aimed to analyze the most factors influencing fertility differentials in Burundi. According to our findings, the total number of children ever born (CEB) ranged from 0 to 15 children by women with a mean of 2.7 children (±2.8 SD). Factors such urban residence, increase in the level of education of both women and husbands, female headed households, no history of infant mortality experience and increasing in age at first marriage or first birth are associated with a low fertility rate while factors such as residence especially in Southern region, both women and husband´s agricultural profession, household poverty, lack of knowledge of any contraceptive methods, non-use of modern contraceptive methods and a number of children ≥ 4 as a family size preference are associated with a high fertility rate.

The negative impact of urban residence on Burundi fertility could be explained by couples using more modern contraceptive methods to reduce family size due to the increased cost of raising a child in urban setting (i.e. food, schooling etc). This association was also reported in several previous studies [2,27,28]. With an annual urbanization rate of 13.7% in 2020, Burundi remains the least urbanized country in Eastern Africa [29]. The Burundi government should invest more in promoting urbanization to accelerate fertility transition. On the other hand, some regions especially the southern ones are associated with a higher fertility than that of Bujumbura Mairie. The latter was the political capital of Burundi before becoming the economic capital in 2019. This implies that it has many advantages over other regions (availability of family planning services, high rate of female schooling, urbanization etc.) in favor of a smaller family size by residents. These findings therefore highlight the need for the Burundi government to focus more on this region to accelerate fertility transition. In addition, our results are consistent with those reported in the 2016-17 BDHS final report [7]. Similarly, our study showed the importance of the male but especially female education in reducing fertility rate. Some previous studies reported similar results: education is widely known to strongly influence women´s fertility by delaying age at first marriage and adoption of favorable behaviors to the use of FP services [30]. According to Zah's study [2], women with at least seven years of education were distinguished from their illiterate counterparts by low fertility. In Burundi, the school attendance rate has increased somewhat over the last decade. However, the overall level remains low for both female and male [23]. Thirty six percent of women and 26% of men are illiterate while 50% of women and 57% of men have only a complete or incomplete primary level education in Burundi [7]. Our results emphasize the need for Burundi policy makers to ensure access to education for all, especially for girls, to accelerate fertility transition.

In line with some previous studies [2,3], our study showed that agricultural profession is associated with a high fertility. Such an association could be due to Burundi agricultural production systems that remain traditional and thus require a larger family workforce. As agriculture is the main source of income for more than 90% of Burundians [10], most families desire a large number of children because of their important contribution to their parents' agricultural activities. Agricultural mechanization could help reduce Burundi fertility level. Our study revealed that household poverty is associated with high fertility. some previous studies [27,31] reported similar findings. Women with no history of infant mortality experience have a lower fertility rate compared to those reported having already lost at least one child. Our results support those of many researchers [16,32,33] who argue that high infant mortality rates are generally associated with high fertility especially in SSA context. In Burundi, such an association could be justified by the fact that the death of a child leads most Burundian couples to have a new birth to replace the deceased child. Moreover, Burundi remains among the countries with the highest infant mortality rate in SSA [15]. Ensuring child survival could therefore help to accelerate fertility transition.

Similarly, women who had their first marriage or birth at an advanced age have significantly lower fertility than those with early marriage or childbearing. Our results are consistent with those of many researchers [27,34,35] who estimate that early marriage not only provides a longer reproductive life but also leads to early childbearing, resulting in a high fertility. Furthermore, early childbearing is a major determinant of large family size and rapid population growth, particularly in countries where contraception is not widely practiced [36]. In Burundi, 23% of the population are adolescents [5], and 8% of women aged 15-19 have already begun childbearing [7]. This underscores the need for the Burundian government to further promote girls´ education to reduce population growth. Also, our study showed that the knowledge and the use of modern contraceptive methods are associated with low fertility. Our results are consistent with those reported in several studies [27,28,37,38]. Nevertheless, modern contraceptive prevalence remains low (23%) and a high proportion of married women (30%) still have unmet need for FP in Burundi [7]. Significant efforts must be made to ensure equitable access to FP services. Finally, our study revealed that as the number of children desired by the family increases so does the risk of high fertility. Our results are consistent with those of Ariho and his collaborator [34] who considered that family size preferences affect the individuals´ fertility behavior particularly decisions about whether or not to use contraceptive methods.

The limitation of our study is that we limited ourselves to the analysis of the correlate variables that were available in the 2016-17 BDHS dataset since our study was a secondary data analysis. Moreover, since our analysis relied on a cross-sectional survey, we found only associations and not causal relationships. Our study’s strength is that this study would be among the first ones to use an analytical approach on nationally representative data to identify the determinants of fertility differentials in Burundi.

## Conclusion

According to our findings, the total number of CEB ranged from 0 to 15 children by women with a mean of 2.7 children (±2.8 SD). Factors such as urban residence, increase in the level of education of both women and husbands, no history of infant mortality experience and increasing in age at first marriage or first birth are associated with a low fertility rate while factors such as residence especially in Southern region, both women and husband´s agricultural profession, household poverty, lack of knowledge of any contraceptive methods and non-use of modern contraceptive methods are associated with a high fertility rate. Actions aimed at promoting education in general especially female education, improving child survival and women´s socio-economic status, agriculture mechanization and increasing number and scope of family planning services could help reduce Burundi fertility rate.

### What is known about this topic

- Studies on fertility determinants are currently plentiful in developed countries, even in some low-income countries such as those in SSA;
- Fertility control remains a major priority for the Burundian government and most of its partners;
- However, few studies on fertility are available to guide their interventions.

### What this study adds

- Our study would be among the first ones to use an analytical approach to identify the factors influencing fertility differentials in Burundi;
- Our study identified other factors influencing fertility differentials (previous experience of infant mortality, husband education and occupation etc.) besides the promotion of FP services and other factors that were already described in the three BDHS reports;
- Our study used a nationally representative sample, which allows the results of this study to be generalized at the national level.
